# Predictive characteristic of simple bone cyst treated with curettage and bone grafting

**DOI:** 10.1186/s12891-015-0797-6

**Published:** 2015-11-14

**Authors:** Pawel Flont, Krzysztof Malecki, Anna Niewola, Zbigniew Lipczyk, Kryspin Niedzielski

**Affiliations:** Clinic of Orthopaedic and Traumatology, Polish Mother’s Memorial Hospital Research Institute, Rzgowska 281/289, 93-338 Lodz, Poland

**Keywords:** Simple bone cyst, Children, Curettage, Recurrences

## Abstract

**Background:**

The efficiency of treating simple bone cyst (SBC) is low. Depending on the choice of treatment, a positive response occurs in 20 to 80 % of cases. These rates are unacceptable, particularly considering they concern the treatment of benign lesions affecting children. Although cyst curettage is one of the first known ways of treating SBC, no precise qualification criteria exists for this procedure. The aim of our study is to identify which type of cyst may be most effectively treated using curettage with grafting.

**Methods:**

A retrospective analysis was performed on 24 patients referred to our clinic for SBC treatment. To identify predictive factors, the group of patients who positively responded to treatment (Neer stages I and II, *n* = 14) were compared with the group in which recurrences occurred (Neer stages III and IV, *n* = 10).

**Results:**

Significantly fewer patients with lesions located in the humerus (chi^2^ = 9.351; *p* <0.05) and without pathological facture at the time of diagnosis (*p* = 0.017) were found in the group with no recurrence. The following radiological parameters were found to vary significantly between groups: cyst area (z = 3.121; *p* < 0.01), cyst index (z = 2.213; *p* <0.05) and cyst diameter ratio (z = 2.202; *p* <0.05). In the group with no recurrences, the mean values of these parameters were found to be lower than in group with poor response to treatment. No statistically significant differences regarding age, sex or type of bone graft (*p* > 0.05) were found. Recurrences were experienced by 10 patients (41.7 %) during the 3-year period after surgery

**Conclusion:**

In the group treated with curettage, associations were identified between worse treatment results and the location in the humerus, pathological fractures at the time of diagnosis, large cyst area, large cyst index and large cyst diameter.

## Background

Simple bone cysts (SBC) are oval, osteolytic, tumor-like lesions with a central location. SBCs occur before the end of bone growth, most commonly in the proximal humerus and proximal femur. The annual prevalence is 0.3 per 100.000 individuals [1].

Although the etiology of the cysts remains unclear, there are many possible explanations for their development, including inflammation, excessive bone resorption or incorrect proto-oncogen expression. In addition, disturbed venous blood outflow from fast growing metaphysis can obstruct intercellular fluid circulation and leads to cyst formation [2–4]. Inner cyst fluid is known to have osteoclastic potential as a result of increased concentration of prostaglandins, interleukins 1 and 6, tumor necrosis factor-alpha and lysosomal enzymes [5–7]. Many authors consider that increased levels of pro-inflammatory cytokines, osteolytic enzymes, together with a higher nitric oxide concentration, confirm that inflammation is a cause of cyst formation [7]. To our knowledge, only two publications report changes in the genome, these being a translocation involving chromosomes 16 and 20 [8, 9]. The authors emphasize the need for further research to confirm frequency of these changes and their etiological relevance.

As etiology is varied, and the choice of treatment strategy depends on etiology, there are many possible approaches to treating SBC. The efficiency of steroid treatment confirms that the disease has an inflammatory basis [10]. Proponents of the venous outflow disturbance theory consider the best treatment to be to drain the cyst, either by drilling, cannulated screw implantation or intramedullary nailing [11, 12]. However, those who believe excessive osteoclast activation to be the cause choose bone grafting procedures using osteoinductive and osteoconductive materials such as demineralized bone matrix, bone marrow or cancellous bone graft [13–15].

SBC treatment efficiency is low. Depending on the choice of treatment, a positive response occurs in 20 to 80 % of cases [16–19]. These rates are unacceptable, particularly considering they concern the treatment of benign bone lesions affecting children and adolescents. Treatment failure is established on the basis of lack of rebuilding progress, recurrences, pathological fractures or growth disturbances.

In general, treatment strategies of SBCs are technically simple with relatively low surgical risk. Certain limits could be the cost of biologically active materials such as demineralized bone matrix or bone morphogenetic protein. When attempting to treat SBC, the surgeon should be prepared to perform several surgical procedures. Accurate treatment selection depends on patient age, location and disease dynamics. The aim of our study is to identify which type of cyst may be most effectively treated using curettage with grafting. The predictive factors for success of this surgical procedure will be determined to identify patients who will gain most benefit from this therapy.

## Methods

A retrospective analysis was performed on 24 patients referred to our clinic for SBC treatment in the period 2005 to 2011. The following study inclusion criteria applied: participant’s age at the time of surgery <18 years, cyst treated with curettage with bone grafting, surgical procedure performed at the study site, and SBC confirmed by pathology (fluid cytology and cyst wall). Cysts treated with other methods (intramedullary stabilisation, bone marrow injection), or cysts located in the spine or the skull were excluded from the study. The demographic characteristics of the subjects enrolled for the study together with cyst locations are presented in Table 1. After identifying the cyst on the X-ray the following radiological parameters were determined:

- number of cyst cavities (uni- or multilocular),
- cyst type, according to Enneking’s scale for benign tumours (latent, active, aggressive) [20],
- cyst area,
- cyst index according to Kaelin and MacEwen [21] (i.e. the cyst area divided by diaphyseal diameter squared),
- cyst diameter ratio (i.e. the extent of the lesion on the longitudinal axis divided by the normal expected diameter of the long bone) [22],
- minimal cortical thickness,
- ratio of the minimal cortical thickness to the cortical thickness in the unaffected part of the diaphysis adjacent to the lesion,
- cyst activity (a cyst located ≤0.5 cm from the growth plate was considered active, whereas all cysts located >0.5 cm from the growth plate were considered latent) [16],
- occurrence of pathological fractures.

**Table 1:** Demographic data for patients with SBCs

Sample size	24
Age at diagnosis — range (years)	3–17
Mean age (years)	11.58
Females (%)	33.33
Males (%)	66.67
Humerus, *n* (%)	6 (25)
Femur, *n* (%)	11 (45.83)
Tibia, *n* (%)	2 (8.33)
Calcaneus, *n* (%)	4 (16.67)
Phalanx, *n* (%)	1 (4.17)

Curettage was performed with the use of a bone curette and a high-speed burr. The adequacy of curettage was ascertained by direct vision and the procedure was performed until damage of the fibrous cyst wall occurred and normal looking bone marrow was seen. A connection with the medullary canal was created. In cases where part of the SBC was close to the epiphyseal plate, only a bone curette was used under direct vision. In ten cases, resection cavities were filled with tibial autografts and in fourteen, with deep-frozen allogeneic bone.

The mean follow-up period was 6.2 years (range three to nine years). The outcomes were evaluated radiologically: X-rays of the affected limb were taken in standard positions every three months after surgery and once a year after cyst resolution. Recurrences were defined according to Neer scale as modified by Chang et al. [23]. Stages I and II signified that the therapy was successful, whereas stages III and IV referred to therapy failure (recurrence). Limb length was assessed according to the AO Foundation guidelines, with a 1.5cm difference for the lower limbs and a 2cm difference for the upper limbs considered clinically relevant.

To identify predictive factors, the group of patients who positively responded to treatment (Neer stages I and II, *n* = 14, 58.3 %, group 1) were compared with the group in which recurrences occurred (Neer stages III and IV, *n* = 10, 41.7 %, group 2).

The χ^2^ test of independence was used to compare the number of qualitative parameters in the study groups and subgroups selected on the basis of different variables; Yates’ correction was used for small samples. As most of the parameters analysed in the study were not normally distributed, the mean values were compared using non-parametric tests: the Mann–Whitney test for two groups and the Kruskal-Wallis test for several independent groups. The study was approved by the Institutional Review Board.

## Results

For further statistical analyze were taken group of patients with no significant differences regarding age (z = 0.016; *p* > 0.05), sex (*p* = 0.327) or kind of graft (*p* = 0.257). The results are presented in Table 2.

**Table 2:** Age, sex and type of bone graft in the study group

	Positive response Neer Stages: I and II	Recurrences Neer Stages: III and IV	Test value	*p*-value
Sample size, *n*	14	10		
Mean age (SD), *years*	11.57 (4.94)	11.6 (2.91)	z = 0.016	*p* > 0.05
Female, *n*	5	3		*p* = 0.327
Male, *n*	9	7		
Allogenic bone graft, *n*	9	5		*p* = 0.257
Autogenous bone graft, *n*	5	5		

Significantly fewer patients with lesions located in the humerus were found in the group with no recurrence (chi^2^ = 9.351; *p* <0.05, Table 3). Another statistically significant difference in both groups is the presence of pathological fractures at the time of diagnosis. The group of patients with no recurrences included a higher proportion of patients without fractures present before the beginning of treatment than the group with recurrences.(*p* = 0.017, Table 3). The following radiological parameters were found to vary significantly between groups: cyst area (z = 3.121; *p* < 0.01), cyst index (z = 2.213; *p* <0.05) and cyst diameter ratio (z = 2.202; *p* <0.05, Table 3). In the group with no recurrences, the mean values of these parameters were found to be lower than in group with poor response to treatment. 

No significant correlations were found regarding location (tibia, femur or other), shaft vs metaphysis, active vs latent lesions, uni- or multilocular lesions, Enneking classification type or such radiological parameters as mean cortical thickness or mean ratio of the minimal cortical thickness to the cortical thickness in the diaphysis (*p* > 0.05, Table 3).

**Table 3:** Morphological and radiological cyst characteristics in the study group

	Positive response	Recurrence	Test value	*p* -value
Sample size	14	10		
Cyst location				
Humerus, *n* (%)	1 (7.14)	5 (50.0)	χ^2^ = 9.351	*p* < *0.05*
Femur, *n* (%)	6 (42.86)	5 (50.0)		
Tibia, *n* (%)	2 (14.29)	0		
Other localistaion [calcaneus phalanx], *n(%)*	5 (35.71)	0		
Cyst location: shaft/metaphysis				
Metaphysis, *n* (%)	7 (50.0)	7 (70.0)		*p* = 0.210
Shaft, *n* (%)	7 (50.0)	3 (30.0)		
Cyst activity				
Active cyst, *n* (%)	4 (28.57)	3 (30.0)		*p* = 0.347
Latent cyst, *n* (%)	10 (71.43)	7 (70.0)		
Number of cavities				
Uninocular, *n* (%)	9 (64.29)	3 (30.0)		*p* = 0.089
Mulitlocular, *n* (%)	5 (35.71)	7 (70.0)		
Pathological fractures at the time of diagnosis				
Yes, *n* (%)	4 (28.57)	8 (80.0)		*p* = *0.017*
No, *n* (%)	10 (71.43)	2 (20.0)		
Enneking stage				
IA, *n* (%)	11 (78.57)	5 (50.0)		*p* = 0.089
IB, *n* (%)	3 (21.43)	4 (40.0)		
IC, *n* (%)	0	1 (10.0)		
Radiological parameters				
Mean cyst area, cm^2^ (SD)	9.99 (7.36)	22.59 (11.55)	z = 3.121	*p* < *0.01*
Mean cyst index of Kaelin and MacEwen (SD)	10.14 (9.74)	19.79 (10.54)	z = 2.213	*p* < *0.05*
Mean cyst diameter ratio (SD)	2.16 (1.72)	3.92 (2.01)	z = 2.202	*p* < *0.05*
Mean minimal cortical thickness, mm^2^ (SD)	0.25 (0.01)	0.19 (0.14)	z = 1.202	*p* > 0.05
Mean ratio of the minimal cortical thickness to the cortical thickness in the diaphysis (SD)	0.61 (0.25)	0.41 (0.27)	z = 1.784	*p* > 0.05

**Fig. 1:**
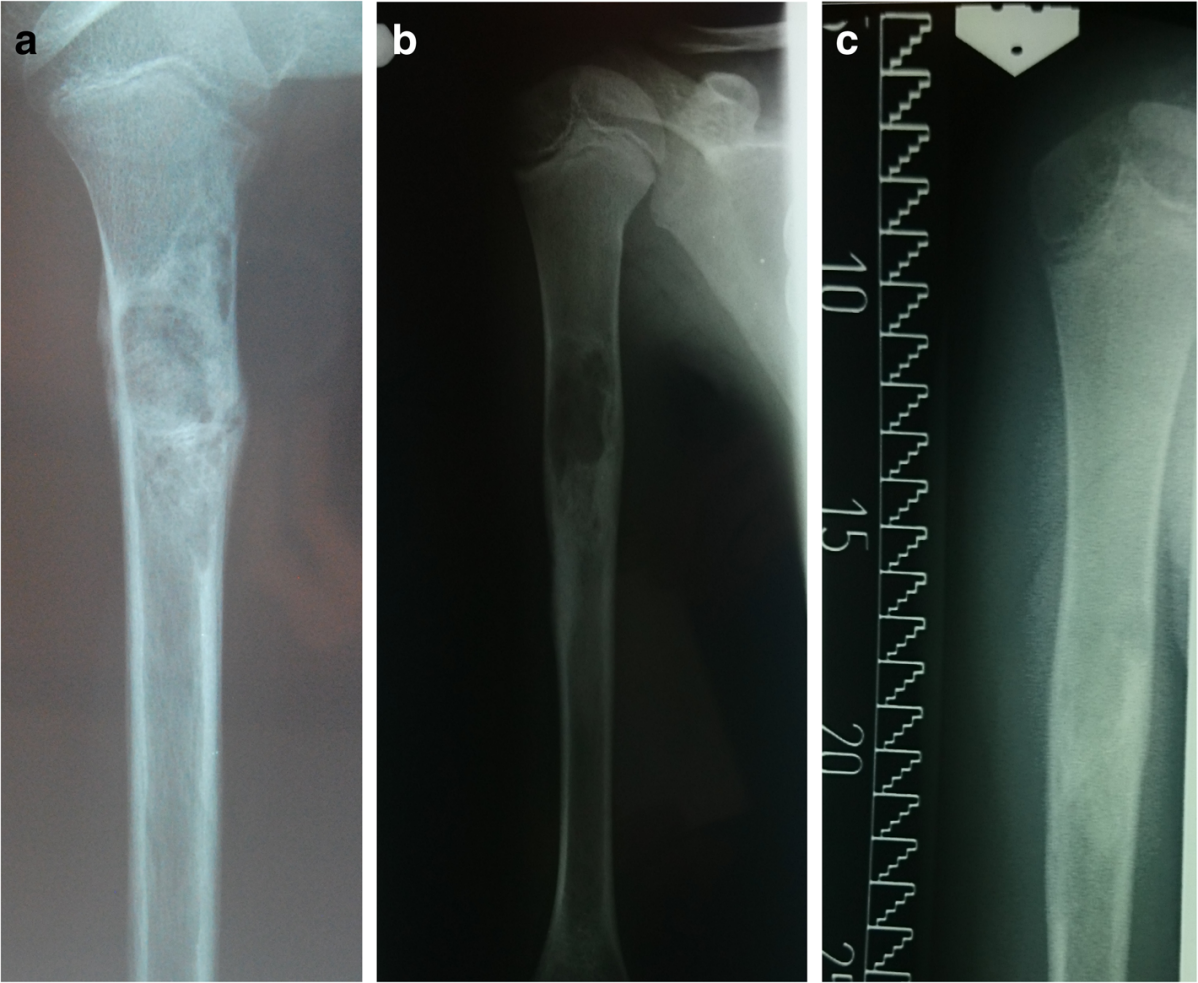
Example of a patient with SBC with recurrence after curettage and bone grafting treated with steroid injection. **a** X-ray of the lesion 3 months postoperatively; (**b**) recurrence at 9 months postoperatively treated with steroid injection; (**c**) final result, 3 years postoperatively. The SBC is healed

**Fig. 2:**
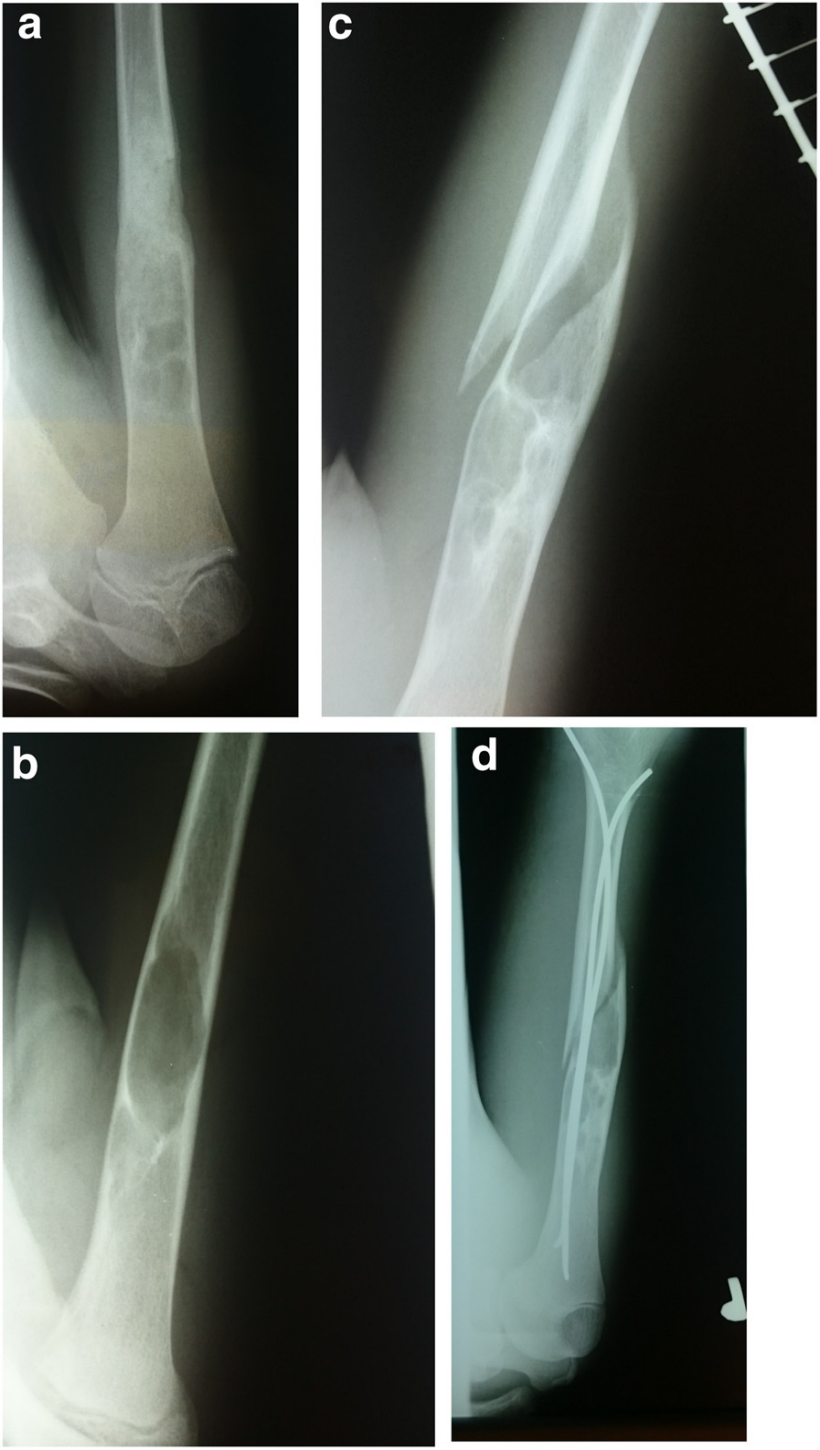
Example of a patient with SBC with recurrence after curettage and bone grafting treated with titanium elastic nails. **a** X-ray of the lesion at 3 months postoperatively; (**b**) at 12 months postoperatively; (**c**) recurrence with pathological fracture; (**d**) X-ray follow-up directly after the titanium elastic nails implantation

Recurrences were experienced by 10 patients (41.7 %) during the 3-year period after surgery. In treating recurrences, the following techniques were used: methylprednisolone injection (7 patients, Fig. 1), en bloc excision followed by transplantation of autogenic fibular grafts (large cysts, 2 patients) and titanium intramedullary nailing (1 patient, Fig. 2). The follow-up revealed limb length discrepancy in 4 patients (16.7 %) and pathological fractures were observed in 4 patients (16,7 %): 2 cases of humeral cysts, 1 case of an intertrochanteric cyst and 1 case of a cyst in the distal tibia.

## Discussion

Until the 1980s, the most commonly performed procedure for the treatment of simple bone cysts was curettage and grafting. This procedure was first reported by Neer in 1966 [16], who demonstrated that complete healing of SBC occurred in only about 50 % of a large group of 130 patients. Recurrences occurred in 15 % of patients treated with this procedure together with additional chemical resection of cyst walls (phenol or zinc chloride), and in 39 % of patients treated with curettage and grafting alone. Patients with poor response to treatment, 26 % of the group, had to undergo further surgery.

In 1986, Campanacci et al. gained complete healing in 46 %, partial healing in 21 % and recurrences in 33 % of patients after curettage with bone grafting. Most of the recurrences were classified for reoperation. The use of different kinds of grafts (auto or allogenic) had no effect on the outcome. The author emphasizes that as free space enables recurrences, the entire lesion lodge should be filled with grafts. However, the grafts shouldn’t be packed too tightly, to avoid hindering revascularization within the cavity [17].

The percentage of failures in the case of curettage is unacceptable, especially considering that it is simply treatment of benign tumor-like lesion in adolescents. The way to lower the recurrence and complication rates is to use an adjuvant procedure such as chemical or physical resection (phenol, hydrogen peroxide, liquid nitrogen or argon beam) [16, 24], which results in a reduced recurrence rate by broadening the zone of tissue necrosis. In 1997, Schreuder published a retrospective work evaluating the outcomes of SBC treatment with curettage, cryosurgery and filling the cyst cavity with allogenic bone grafts. After mechanical curettage of the cyst, the author filled the cavity with liquid nitrogen, which was washed out with warm saline in the next step. Complete healing was achieved by 50 % of patients, partial healing in 38 % and recurrences requiring reoperation in 12 % [24].

A meta-analysis of 12 studies published by Schreuder was used to compare recurrences after curettage in our study group to outcomes found in literature [24]. The mean number of recurrences is 27.4 % of patients, with the highest percentages of failure being reported by Bovill et al. (46.6 %) [18] and Mylle et al. (47.6 %) [19], and the lowest number of recurrences by Peltier et al. (7.7 %) [25]. The percentage of recurrences in our study is rather high (41.7 %). This may be a result of the fact that mean follow-up time is quite long (6.2 years), with a minimal follow-up time of 3 years. Some of the reports analysed by Schreuder in the meta-analysis note a minimal follow-up time 1–2 years, which is a rather short period of time to observe possible recurrences and rebuilding of synthetic osteoconductive materials [17, 26]. Also, the evaluation of cyst rebuilding on the basis of the Neer scale, the most commonly-used framework, is not clearly defined. Neer fails to clearly define the meaning of “partial healing” and the correct criteria of recurrence and therapy failure. Hence, different values for cyst area or minimal cortical thickness were chosen to determine therapy failure. To make the present study more objective, the Neer scale was used with Chang’s modification, which defines partial cyst healing as taking place if the osteolytic lesion takes less than 50 % the diameter of the bone, with enough cortical thickness to prevent fracture [23]. Our study assumes the minimal cortical thickness qualifying to partial healing is 1 mm. This method of evaluation allows the results qualified in our work as recurrence to be compared with partial healing in other works. This study tightens, or rather systematizes, the criteria for complete healing in response to of patient expectations. Nowadays, even children regularly take part in sport and are very physically active. As complete healing means, for both patient and parents, the ability to return to earlier activity before diagnosis, the mechanical properties of bone need to be fully recovered.

Few studies have attempted to evaluate factors predictive of response to different modes of SBC treatment. After curettage and bone grafting, Neer et al. observed a higher percentage of recurrences in cysts located in the proximal parts of the humerus and distal part of the femur and better results in the tibia and fibula [16]. Campanacci et al. conclude that recurrence rate was higher in case of active cysts after curettage [17]. Schreuder in his work evaluating curettage does not report significant correlations between the risk of recurrence and age, sex, cyst volume, type of previous treatment procedures or pathological fractures [24]. Mik does not note any statistically significant correlation between recurrence ratio and age (*p* = 0.055), pathological fracture (*p* > 0.1) or cyst activity (*p* = 0.41). The mean age varies significantly between the groups: 8.7 versus 11.3 years. Therefore, with the probability slightly lower than usual, it can be assumed that younger children are less likely to experience satisfactory outcomes [27].

## Conclusions

In the group treated with curettage, correlations were identified between worse treatment results and the location in the humerus, pathological fractures at the time of diagnosis, large cyst area, large cyst index and large cyst diameter. Such relevance has not been previously reported, because only few authors have analyzed the reasons for recurrence. In the case of previous pathological fracture, the cavity of the cyst becomes irregular and is divided by cortical layers, which makes curettage technically harder to perform and sometimes makes the excision of complete cyst borders impossible. In the humerus, the SBC are usually larger with thinner walls than those in other locations. Due to their asymptomatic course, they are diagnosed after first fracture. In our opinion, the higher recurrence rates observed following this more technically demanding curettage can be attributed to the large size, thin wall and irregularity of cortical layers after fracture. The presence of statistically significant predictive factors is due to the high percentage of patients with poor outcomes in our study group. Additionally, as many as 13 factors were compared in our work; No such detailed comparison has been previously reported.

## References

[1] Zehetgruber H, Bittner B, Gruber D, Krepler P, Trieb K, Kotz R, Dominkus M (2005). Prevalence of aneurysmal and solitary bone cysts in young patients. Clin Orthop Relat Res.

[2] Cohen J (1960). Simple bone cysts. Studies of cyst fluid in six cases with a theory of pathogenesis. J Bone Joint Surg Am.

[3] Chigira M, Maehara S, Arita S, Udagawa E (1983). The aetiology and treatment of simple bone cysts. J Bone Joint Surg Br.

[4] Watanabe H, Arita S, Chigira M (1994). Aetiology of a simple bone cyst. Case Report.

[5] Shindell R, Huurman WW, Lippiello L, Connolly JF (1989). Prostaglandin levels in unicameral bone cysts treated by intralesional steroid injection. J Pediatr Orthop.

[6] Komiya S, Minamitani K, Sasaguri Y, Hashimoto S, Morimatsu M, Inoue A (1993). Simple bone cyst. Treatment by trepanation and studies on bone resorptive factors in cyst fluid with a theory of its pathogenesis. Clin Orthop Relat Res.

[7] Komiya S, Kawabata R, Zenmyo M, Hashimoto S, Inoue A (2000). Increased concentrations of nitrate and nitrite in the cyst fluid suggesting increased nitric oxide synthesis in solitary bone cysts. J Orthop Res..

[8] Richkind KE, Mortimer E, Mowery-Rushton P, Fraire A (2002). Translocation (16;20)(p11.2;q13). sole cytogenetic abnormality in a unicameral bone cyst. Cancer Genet Cytogenet.

[9] Vayego SA, De Conti OJ, Varella-Garcia M (1996). Complex cytogenetic rearrangement in a case of unicameral bone cyst. Cancer Genet Cytogenet.

[10] Scaglietti O, Marchetti PG, Bartolozzi P (1982). Final results obtained in the treatment of bone cysts with methylprednisolone acetate (depo-medrol) and a discussion of results achieved in other bone lesions. Clin Orthop Relat Res.

[11] Shinozaki T, Arita S, Watanabe H, Chigira M (1996). Simple bone cysts treated by multiple drill-holes. 23 cysts followed 2–10 years. Acta Orthop Scand.

[12] Abdel-Wanis ME, Tsuchiya H, Uehara K, Tomita K (2002). Minimal curettage, multiple drilling, and continuous decompression through a cannulated screw for treatment of calcaneal simple bone cysts in children. J Pediatr Orthop.

[13] Lokiec F, Ezra E, Khermosh O, Wientroub S (1996). Simple bone cysts treated by percutaneous autologous marrow grafting. A preliminary report. J Bone Joint Surg Br.

[14] Killian JT, Wilkinson L, White S, Brassard M (1998). Treatment of unicameral bone cyst with demineralized bone matrix. J Pediatr Orthop.

[15] Altermatt S, Schwöbel M, Pochon JP (1992). Operative treatment of solitary bone cysts with tricalcium phosphate ceramic. A 1 to 7 year follow-up. Eur J Pediatr Surg.

[16] Neer CS, Francis KC, Marcove RC, Terz J, Carbonara PN (1966). Treatment of unicameral bone cyst. A follow-up study of one hundred seventy-five cases. J Bone Joint Am.

[17] Campanacci M, Capanna R, Picci P (1986). Unicameral and aneurysmal bone cysts. Clin Orthop Relat Res.

[18] Bovill DF, Skinner HB (1989). Unicameral bone cysts. A comparison of treatment options. Orthop Rev.

[19] Mylle J, Burssens A, Fabry G (1992). Simple bone cysts. A review of 59 cases with special reference to their treatment. Arch Orthop Trauma Surg.

[20] Enneking WF (1986). A system of staging musculoskeletal. Clin Orthop Relat Res.

[21] Kaelin AJ, MacEwen GD (1989). Unicameral bone cysts. Natural history and the risk of fracture. Int Orthop.

[22] Kanellopoulos AD, Mavrogenis AF, Papagelopoulos PJ, Soucacos PN (2007). Elastic intramedullary nailing and DBM-bone marrow injection for the treatment of simple bone cysts. World J Surg Oncol.

[23] Chang CH, Stanton RP, Glutting J (2002). Unicameral bone cysts treated by injection of bone marrow or methylprednisolone. J Bone Joint Surg Br.

[24] Schreuder HW, Conrad EU, Bruckner JD, Howlett AT, Sorensen LS (1997). Treatment of simple bone cysts in children with curettage and cryosurgery. J Pediatr Orthop.

[25] Peltier LF, Jones RH (1978). Treatment of unicameral bone cysts by curettage and packing with plaster-of-Paris pellets. J Bone Joint Surg Am.

[26] Oppenheim WL, Galleno H (1984). Operative treatment versus steroid injection in the management of unicameral bone cysts. J Pediatr Orthop.

[27] Mik G, Arkader A, Manteghi A, Dormans JP (2009). Results of a minimally invasive technique for treatment of unicameral bone cysts. Clin Orthop Relat Res.

